# Inhibition of Cdk2 activity decreases Aurora-A kinase centrosomal localization and prevents centrosome amplification in breast cancer cells

**DOI:** 10.3892/or.2013.2313

**Published:** 2013-02-27

**Authors:** ALEXEY A. LEONTOVICH, JEFFREY L. SALISBURY, MASSIMILIANO VEROUX, TIZIANO TALLARITA, DANIEL BILLADEAU, JAMES McCUBREY, JAMES INGLE, EVANTHIA GALANIS, ANTONINO B. D’ASSORO

**Affiliations:** 1 Department of Biochemistry and Molecular Biology, Mayo Clinic, College of Medicine, Rochester, MN, USA; 2 Department of Biomedical Statistics and Informatics, Mayo Clinic, College of Medicine, Rochester, MN, USA; 3 Department of Medical Oncology, Mayo Clinic, College of Medicine, Rochester, MN, USA; 4 Brody School of Medicine at East Carolina University, Greenville, NC, USA; 5 Department of Surgery, Transplantation and Advanced Technologies, Organ Transplant Unit and Vascular Surgery, University of Catania, Catania, Italy

**Keywords:** breast cancer, centrosome amplification, Aurora-A, Cdk2, genotoxic stress

## Abstract

Centrosome amplification plays a key role in the origin of chromosomal instability (CIN) during cancer development and progression. In this study, MCF-7 breast cancer cell lines harboring abrogated p53 function (vMCF-7^DNp53^) were employed to investigate the relationship between induction of genotoxic stress, activation of cyclin-A/Cdk2 and Aurora-A oncogenic signalings and development of centrosome amplification. Introduction of genotoxic stress in the vMCF-7^DNp53^ cell line by treatment with hydroxyurea (HU) induced centrosome amplification that was mechanistically linked to Aurora-A kinase activity. In cells carrying defective p53, the development of centrosome amplification also occurred following treatment with another DNA damaging agent, methotrexate. Importantly, we demonstrated that Aurora-A kinase-induced centrosome amplification was mediated by Cdk2 kinase since molecular inhibition of Cdk2 activity by SU9516 suppressed Aurora-A centrosomal localization and consequent centrosome amplification. In addition, we employed vMCF-7^DRaf-1^ cells that display high levels of endogenous cyclin-A and demonstrated that molecular targeting of Aurora-A by Alisertib reduces cyclin-A expression. Taken together, these findings demonstrate a novel positive feed-back loop between cyclin-A/Cdk2 and Aurora-A pathways in the development of centrosome amplification in breast cancer cells. They also provide the translational rationale for targeting ‘druggable cell cycle regulators’ as an innovative therapeutic strategy to inhibit centrosome amplification and CIN in breast tumors resistant to conventional chemotherapeutic drugs.

## Introduction

The progression of aggressive breast cancer is characterized by genomic instability leading to multiple genetic defects, phenotypic heterogeneity, chemoresistance and poor outcome (1,2). An imbalance between oncogene and tumor suppressor activities plays an important role in the onset of breast cancer through the inactivation of G_1_/S and/or G_2_/M cell cycle checkpoints, which normally ensure the orderly progression of cell cycle events (3). In normal cells, checkpoint activation in response to DNA damage is mediated by p53 activation and inhibition of Cdk2 activity leading to cell cycle arrest (4–6). The centrosome has been implicated in the pathogenesis of cancer through the development of multipolar mitotic spindles leading to chromosomal instability and tumor cell heterogeneity (7,8). The centrosome is the major microtubule-organizing center of the cell and is duplicated once during a normal cell cycle to give rise to two centrosomes that function as the spindle poles during mitosis (3). Therefore, tight coordination between centrosome duplication and DNA replication cycles is essential to ensure equal segregation of sister chromatids during cell division. In cancer, loss of coordination between the centrosome and DNA cycles leads to centrosome amplification, increased frequency of multipolar mitoses, and consequent chromosomal instability (5,6,9,10). p53 and cyclin-A/Cdk2 play an important role in coordinating centrosome duplication with cell cycle events. In mouse model studies, loss of p53 by gene targeting and gain-of-function p53 mutations resulted in the development of centrosome amplification and aberrant mitoses (5,6,11). Furthermore, cyclin-A has been demonstrated to be a key regulator of the centrosome cycle (12,13), and p53 mutations associated with cyclin-A overexpression synergistically increase the frequency of centrosome defects (14). Aurora-A mitotic kinase is a critical Ser/Thr protein kinase that also controls centrosome maturation and duplication and regulates spindle formation for appropriate chromosomal segregation during normal mitosis (15). In cancer cells, overexpression of Aurora-A kinase promotes centrosome amplification and chromosomal instability (CIN), thus conferring tumor cell heterogeneity associated with acquired drug resistance and poor outcome (16). Aurora-A is overexpressed in human breast tumors and is associated with an invasive basal-like phenotype and poor prognosis (17). Aurora-A exerts a direct effect on oncogenic transformation *in vitro* and *in vivo* through increased p53 degradation and inhibition of apoptosis through activation of the PI3K/AKT pathway leading to chemoresistance (18). In human breast cancer, the mechanistic relationship between deregulated activity of the cyclin-A/Cdk2 complex and Aurora-A kinase in the induction of centrosome amplification has not been investigated. To establish the molecular mechanisms linking genotoxic stress, G_1_/S checkpoint and Aurora-A kinase activity to the centrosome duplication cycle, we studied the effect of drugs inducing genotoxic stress in breast tumor-derived cell lines with abrogated p53 function as previously described (5,6). Our results demonstrated that induction of genotoxic stress induces centrosome amplification through stabilization and activation of Aurora-A kinase mediated by Cdk2 oncogenic signaling in breast cancer cells.

## Materials and methods

### Human breast cancer cell lines

The human breast cancer cell line MCF-7 was obtained from ATCC (Manassas, VA, USA). The MCF-7 cells carrying a dominant-negative p53 mutant (vMCF-7^DNp53^) or overexpressing a constitutive active Raf-1 oncoprotein (vMCF-7^DRaf-1^) were generated as previously described (5,6,19,20).

### Induction of genotoxic stress

To investigate the relationship between centrosome amplification and G_1_/S checkpoint activation, cell lines were plated at a density of 3×10^5^. After 48 h, cells were treated with 2 mM hydroxyurea (HU) or 1 μM methotrexate for 48 h to induce genotoxic stress and centrosome amplification.

### Treatment of cancer cells with small-molecule inhibitors of Cdk2 and Aurora-A

To inhibit Cdk2 or Aurora-A kinase activity, cancer cells were treated with 1 μM SU9516 or 1 μM Alisertib, and the resulting cellular phenotype was analyzed by immunofluorescence and immunoblotting.

### Indirect immunofluorescence and immunoblotting

For indirect immunofluorescence and protein expression analyses, breast cancer cells were treated as previously described (5,6,19,20). Antibodies employed in this study were the following: Aurora-A (Cell Signaling Technology, Inc., Beverly, MA, USA); cyclin A (Santa Cruz Biotechnology, Inc., Santa Cruz, CA, USA) and β-actin (Sigma, St. Louis, MO, USA). Centrin antibody (20H5) was kindly provided by Dr Salisbury’s Laboratory (Mayo Clinic, Rochester, MN, USA).

### Construction of the shRNA Aurora-A vector

The PSSH1 shRNA suppression plasmid contains the H1 RNA polymerase III-dependent promoter for the generation of shRNA molecules. shRNA oligos directed against the 39 UTR of Aurora A (TAGGGATTTGCTTGG-GATA) were annealed and cloned into the *Bgl*II/*Hin*dIII cloning site at the 3′ end of the RNA polymerase III-dependent H1 RNA promoter driven vector. Clones containing the insert were identified and sequenced to ensure fidelity.

## Results

**Figure 1:**
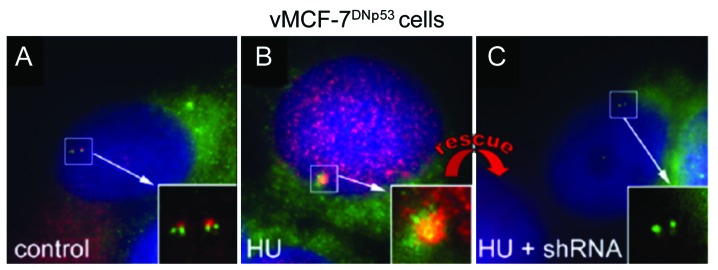
Induction of centrosome amplification following genotoxic stress. (A) Immunofluorescence analysis showing duplicated centrosomes in vMCF-7^DNp53^ cells. (B) Centrosome amplification after hydroxyurea (HU) treatment in vMCF-7^DNp53^ cells. (C) Inhibition of centrosome amplification after HU and shRNA Aurora-A treatment. The centrosome protein centrin was labeled in green, the mitotic kinase Aurora-A was labeled in red and DNA was labeled in blue with Hoechst dye.

To establish the mechanistic linkage between Cdk2 and Aurora-A kinase oncogenic signalings in the induction of centrosome amplification, we employed MCF-7 breast cancer cells harboring abrogated p53 function (MCF-7^DNp53^). The centrosome phenotype was characterized employing antibodies directed against the centrosomal protein centrin and the mitotic kinase Aurora-A. Treatment of MCF-7^DNp53^ cells with HU for 48 h resulted in centrosome amplification characterized by centriole overduplication (Fig. 1A) as previously described (5). Importantly, the amplified centrosomes displayed robust Aurora-A co-localization suggesting that Aurora-A kinase activity may induce centrosome amplification after genotoxic stress (Fig. 1B). To investigate whether Aurora-A kinase activity plays a direct role in the induction of centrosome amplification, we treated MCF-7^DNp53^ cells with HU and shRNA targeting Aurora-A for 48 h. Following knockdown of Aurora-A, centrosome amplification failed to occur after genotoxic stress, and remarkably a normal centrosome phenotype was rescued in MCF-7^DNp53^ cells (Fig. 1C). These results demonstrate the causal role of Aurora-A kinase activity in the induction of centrosome amplification following genotoxic stress.

**Figure 2:**
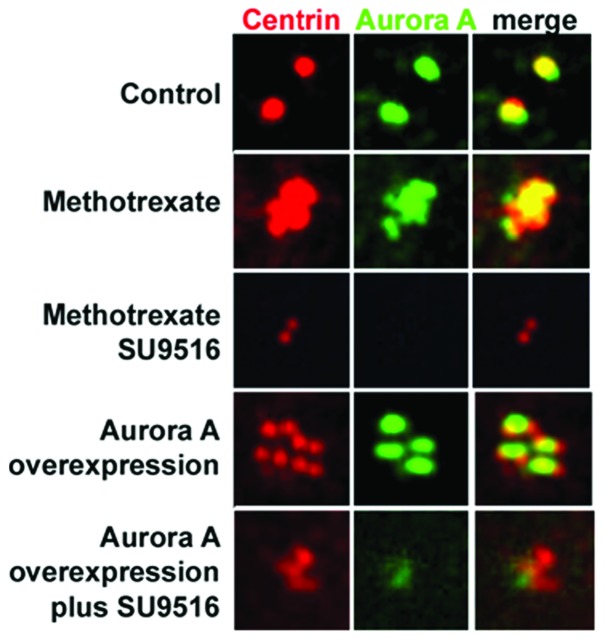
Inhibition of Cdk2 kinase activity restores a normal centrosome phenotype. Immunofluorescence analysis showing a centrosome phenotype in vMCF-7^DNp53^ and vMCF-7^DNp53^-overexpressing Aurora-A cancer cells following treatment with methotrexate and SU9516. The centrosome protein centrin was labeled in red and the mitotic kinase Aurora-A was labeled in green.

**Figure 3:**
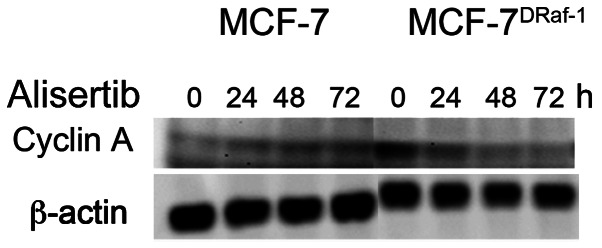
Inhibition of Aurora-A kinase activity reduces cyclin-A expression. Immunoblot analysis of parental and MCF-7 cells overexpressing a constitutive active Raf-1 oncoprotein (vMCF-7^DRaf-1^) showing that molecular targeting of Aurora-A with Alisertib reduces cyclin A expression in vMCF-7^DRaf-1^ cells.

Next, we investigated the mechanistic linkage between Cdk2 and Aurora-A kinase activity in the induction of centrosome amplification following genotoxic stress in MCF-7^DNp53^ and parental MCF-7 cells. We previously demonstrated that abrogation of p53 function leads to deregulated activity of the cyclin-A/Cdk2 complex resulting in induction of centrosome amplification (5,6). In this study, we established an *in vitro* functional assay where MCF-7^DNp53^ and parental MCF-7 cells were treated with methotrexate, a genotoxic agent commonly employed in the adjuvant setting of breast cancer. In order to determine the concentration of methotrexate that will inhibit DNA replication and induce genotoxic stress, we performed dose response and time course experiments with MCF-7 and MCF-7^DNp53^ cells. Our experiments established that incubation for 48 h with 1 μM methotrexate induced a G_1_/S arrest of the cell cycle by FACS analysis (data not shown). To determine the effect of methotrexate on the development of centrosome amplification and Aurora-A centrosomal localization, we incubated MCF-7 and MCF-7^DNp53^ cells with 1 μM methotrexate for 48 h and analyzed the centrosome phenotype using antibodies directed against the proteins centrin and Aurora-A. As previously demonstrated, MCF-7 cells retained a normal centrosome phenotype while vMCF-7^DNp53^ cells developed centrosome amplification (only centrosomes from the vMCF-7^DNp53^ cells are shown in Fig. 2). Genotoxic stress-induced centrosome amplification was associated with an increase in Aurora-A centrosomal localization as we already demonstrated in Fig. 1B. This effect of methotrexate on centrosome amplification and Aurora-A centrosomal localization was blocked by incubation for 48 h with the small-molecule inhibitor of Cdk2 activity, SU9516 (Fig. 2). These results indicated that Aurora-A kinase activity is downstream to Cdk2 signaling in the induction of centrosome amplification. To investigate at the mechanistic level the role of Cdk2 kinase in the activation of Aurora-A-induced centrosome amplification, we engineered vMCF-7^DNp53^ cells overexpressing an Aurora-A lentivector (vMCF-7^DNp53^/Aurora-A). vMCF-7^DNp53^/Aurora-A cells displayed centrosome amplification without induction of genotoxic stress, confirming the causal role of aberrant Aurora-A kinase activity in inducing centriole over-duplication (Fig. 2). Importantly, treatment of vMCF-7^DNp53^/Aurora-A cells with 1 μM SU9516 for 48 h reduced Aurora-A centrosomal localization that was linked to suppression of centrosome amplification (Fig. 2). Since we previously demonstrated that Cdk2 controls the centrosome duplication cycle through interaction with cyclin-A (5), we employed a variant MCF-7 cell line overexpressing a constitutive active Raf-1 oncoprotein (vMCF-7^DRaf-1^) that displays high levels of endogenous cyclin-A (20). To investigate the presence of a positive feed-back loop between cyclin-A/Cdk2 and Aurora-A oncogenic signalings, we treated MCF-7 and vMCF-7^DRaf-1^ cells with a novel Aurora-A inhibitor (Alisertib) to assess the expression of cyclin-A (Fig. 3). Importantly, treatment with Alisertib induced the reduction in cyclin-A expression in vMCF-7^DRaf-1^ cells demonstrating that Aurora-A kinase may modulate cyclin-A/Cdk2 activity during cell cycle progression. Taken together, these findings demonstrate at the mechanistic level a novel interplay between cyclin-A/Cdk2 and Aurora-A oncogenic signalings in the development of centrosome amplification in breast cancer cells.

## Discussion

Deregulation of cell cycle checkpoints, centrosome amplification and induction of CIN are hallmarks of breast cancer (10). CIN represents a major problem in the management of breast cancer patients due to the generation of tumor clonal heterogeneity, which in turn may facilitate the development of chemoresistance and tumor progression (21). However, molecular mechanisms associated with the development of CIN are poorly understood in breast cancer. Several studies suggest that deregulation of the centrosome cycle leading to centrosome amplification may lead to the development of CIN through the formation of multipolar mitotic spindles and unequal chromosome segregation (3,10,22). The tumor-suppressor gene p53, mutated in >50% of human cancers (23), plays an important role in the maintenance of centrosome homeostasis since loss of p53 function can lead to centrosome defects (5,6). Furthermore, the Cdk2/cyclin-A complex has also been implicated in the control of centrosome duplication (5,6,12), and changes in their expression correlates with centrosome amplification (24), suggesting an interplay between the p53 and cdk2/cyclin-A pathways in coordinating centrosome duplication with other cell cycle events. Moreover, the centrosome duplication cycle is also regulated by the mitotic kinase Aurora-A (25). Aberrant Aurora-A kinase activation induces centrosome amplification and CIN in solid tumors (16). However, whether or not deregulated function of the cyclin-A/Cdk2 complex induces centrosome amplification through aberrant activation of Aurora-A kinase has not been established. In the present study, we investigated the mechanistic linkage between cyclin-A/Cdk2 and Aurora-A oncogenic signalings in the development of centrosome amplification in human breast cancer cell models. We employed vMCF-7^DNp53^ cells that display a deregulated activity of the cyclin-A/Cdk2 complex and develop centrosome amplification after genotoxic stress (5,6). Following induction of genotoxic stress, vMCF-7^DNp53^ cells developed centrosome amplification that was functionally linked to an increase in Aurora-A centrosomal localization. Aurora-A played a causal role in the induction of centrosome amplification, since suppression of Aurora-A expression restored a normal centrosome phenotype. Importantly, we demonstrated that Aurora-A kinase-induced centrosome amplification was mediated by Cdk2 activity since molecular targeting of Cdk2 suppressed Aurora-A centrosomal localization and the consequent centrosome amplification. Moreover, we employed vMCF-7^DRaf-1^ cells that display high levels of endogenous cyclin-A, and we demonstrated that molecular targeting of Aurora-A reduces cyclin-A expression. In conclusion, these findings highlight a novel positive feedback loop between cyclin-A/Cdk2 and Aurora-A pathways in the development of centrosome amplification in breast cancer cells. They also provide the operational rationale for targeting ‘druggable cell cycle regulators’ as an innovative therapeutic strategy to inhibit centrosome amplification and CIN in breast tumors resistant to conventional chemotherapeutic drugs.
